# Cancer and cardiovascular-related perceived risk in a diverse cancer center catchment area

**DOI:** 10.1007/s10552-022-01560-3

**Published:** 2022-03-11

**Authors:** Laura C. Pinheiro, Orysya Soroka, Dominic Razon, Rosio Ramos, Francesse Antoine, Andrew J. Dannenberg, Monika Safford, Stephen J. Peterson, Rulla M. Tamimi, David M. Nanus, Erica Phillips

**Affiliations:** 1 Division of General Internal Medicine, Department of Medicine, Weill Cornell Medicine/NewYork–Presbyterian, 420 East 70th Street, Box 331, New York, NY 10021 USA; 2 Research Business Management, Weill Cornell Medicine/NewYork–Presbyterian, New York, NY USA; 3 Department of Medicine, Weill Cornell Medical College, New York, NY USA; 4 Division of General Internal Medicine, Department of Medicine/NewYork Presbyterian, Brooklyn Methodist Hospital, Brooklyn, NY USA; 5 Division of Population Health Science, Weill Cornell Medicine/NewYork–Presbyterian, New York, NY USA; 6 Division of Hematology and Medical Oncology, Department of Medicine, Weill Cornell Medicine/NewYork-Presbyterian Hospital, New York, NY USA; 7 Sandra and Edward Meyer Cancer Center, Weill Cornell Medicine/NewYork–Presbyterian, New York, NY USA

**Keywords:** Risk perception, Cancer, Cardiovascular disease, Healthy behaviors

## Abstract

**Purpose:**

Despite cancer and cardiovascular disease (CVD) sharing several modifiable risk factors, few unified prevention efforts exist. We sought to determine the association between risk perception for cancer and CVD and engagement in healthy behaviors.

**Methods:**

Between May 2019 and August 2020, we conducted a cross-sectional survey of adults ≥ 40 years residing in Brooklyn neighborhoods with high cancer mortality. We considered one’s perceived risk of cancer and CVD compared to age counterparts as the primary exposures. The primary study outcome was a weighted health behavior score (wHBS) composed of 5 domains: physical activity, no obesity, no smoking, low alcohol intake, and healthy diet. Modified Poisson regression models with robust error variance were used to assess associations between perceived risk for cancer and CVD and the wHBS, separately.

**Results:**

We surveyed 2448 adults (mean [SD] age, 61.4 [12.9] years); 61% female, 30% Non-Hispanic White, and 70% racial/ethnic minorities. Compared to their age counterparts nearly one-third of participants perceived themselves to be at higher CVD or cancer risk. Perceiving higher CVD risk was associated with an 8% lower likelihood of engaging in healthy behaviors (RR 0.92; 95% CI 0.86–0.99). Perceiving greater cancer risk was associated with a 14% lower likelihood of engaging in healthy behaviors (RR 0.86; 95% CI 0.79–0.95). The association between cancer risk and wHBS attenuated but remained significant (aRR 0.90; 95% CI 0.82–0.98) after adjustment.

**Conclusion:**

Identifying high-risk subgroups and intervening on shared risk behaviors could have the greatest long-term impact on reducing CVD and cancer morbidity and mortality.

**Supplementary Information:**

The online version contains supplementary material available at 10.1007/s10552-022-01560-3.

## Introduction

Cancer and cardiovascular disease (CVD) are the two leading causes of death worldwide [1]. Commonly considered as two separate disease entities [2, 3], cancer and CVD share modifiable risk factors, pathophysiological mechanisms [4], and often coexist in the same person [5]. In a report by the American Heart Association, adherence to four (not smoking, moderate physical activity, normal body weight, and eating a healthy diet) of seven ideal health behaviors was associated with a significantly lower cancer incidence [2]. Conversely, adherence to cancer prevention guidelines for body weight, diet, physical activity, and alcohol intake resulted in lower rates of CVD among older, non-smoking adults [6]. As such, there is evidence to support a unified approach in assessing risk and preventative efforts of both conditions, yet such an approach has not been widely implemented.

According to health behavior models, knowledge of the negative health consequences of behavior is necessary for behavior change [7, 8]. However, knowledge alone is not sufficient to promote behavior change. Inaccurate risk perceptions can contribute to undesirable health consequences through a lack of adoption and maintenance of preventative health behaviors [9]. While there is a rich literature evaluating the individual impact of cancer or CVD risk perception on changes in behavior [10–12], to our knowledge, no studies have examined their impact together on shared behavioral risk factors in a diverse population. We sought to describe the relationship between an individual’s perceived risk of cancer and their maintenance of preventative health behaviors and, separately, the relationship between an individual’s perceived risk of CVD and their maintenance of the same preventative health behaviors.

## Methods

### Design and setting

This cross-sectional survey study was designed to elucidate adults’ perception of risk for cancer and CVD and to determine if differences in risk perception are associated with the likelihood of engaging in preventive behaviors. We hypothesized that individuals with a higher perceived risk of either cancer or CVD would report engaging in more salutary health behaviors.

We focused on five Brooklyn neighborhoods (Bedford-Stuyvesant, Coney Island, Crown Heights, East Flatbush, and Flatbush/Midwood) where ≥ 20% of the annual cancer cases in 2017 were registered within our health care system. At least one of the top five cancers amendable to early detection (breast, cervix, colon, lung, and prostate) was a leading cause of premature (before age 65) death in the respective neighborhood.

### Eligibility of participants

To be eligible for the survey, adults had to be ≥ 40 years and reside in one of the target neighborhoods based on (1) zip codes associated with their listed telephone number or (2) self-reported zip codes at the time of venue-based sampling. Zip codes were verified at the beginning of the survey. This age range was selected based on most cancer screening recommendations, except cervical, beginning in middle-aged adults. We oversampled individuals with low socioeconomic status (< $35,000 annual household income), as it is associated with an increased incidence of cancer and late-stage diagnosis. [13].

### Survey procedures

We partnered with the Survey Research Institute at Cornell University (Ithaca, NY) to conduct a multimodal survey. A proportional quota sampling frame based on racial, ethnic, and socioeconomic demographics of the five neighborhoods from the 2010 census was used to survey 2500 adults (1500 by landline phone/web and 1000 in-person).

A customized randomly ordered list of households where at least one adult (≥ 40 years) resided in the home and approximately half had incomes of ≤ $34,999 was purchased from Marketing Systems Group. The median household income in Brooklyn in 2017 was $56,942, which was 17% less than the median annual income of $68,486 across the entire state of New York [14]. Participants contacted by telephone had the option to complete the survey via the web. Telephone calls were conducted between 2 May 2019 and 15 September 2019. The survey was conducted in English and Spanish. All participants were offered a $15 gift card for their time.

Venue-based survey sampling was conducted between October 2019 and March 2020. Trained bi-lingual staff conducted the survey anonymously on tablets in English, Spanish, Mandarin, and Russian. Due to the COVID-19 pandemic, venue-based sampling was suspended in mid-March 2020 after surveying 787 participants. The remaining 213 surveys were completed by telephone. A description of venues and numbers recruited from each location can be found in the table in the supplemental materials (Supplementary Table 1). The study was approved by the Institutional Review Board of Weill Cornell Medicine.

### Survey instrument

The survey was guided by the Health Belief Model [15] which describes the beliefs that lead people to prevent, screen for, or control an illness. In this framework, an individuals’ perceived susceptibility (risk perception) about a condition influences their behaviors and actions. Our current analyses focus on the relationship between perceived susceptibility and individual lifestyle behaviors.

The 50-item survey expanded upon components of the Health Information National Trends Survey (HINTS) [16], a biennial, cross-sectional survey that collects nationally representative data about the public’s use of general health and cancer-related information. The 50 questions in our survey covered six domains: (1) health status, (2) health care access, (3) cancer and CVD shared risk behaviors, (4) cancer screening, (5) perceived risk and fatalistic beliefs about cancer and CVD, and (6) demographics and social determinants of health.

### Primary outcome

Using a selection of the American Heart Association's (AHA) Life’s Simple 7 [17] and American Cancer Society guideline [18] on nutrition and physical activity for cancer prevention, we developed a health behavior score (HBS) a priori consisting of 5 health behaviors: cigarette smoking, body weight, physical activity, healthy eating, and alcohol use (Table 1). Each of the five behaviors had three response options: 0 (not meeting recommendations at all, e.g., current smoker); 1 (partial adherence to recommendations, e.g., former smoker); and 2 (full adherence to recommendations, e.g., never smoker). The HBS is the sum of the responses for each of the five behaviors and ranges from 0 (does not adhere) to 10 for (fully adherent). We calculated a weighted HBS (wHBS), which assigned equal weights (1/6) for fruit and vegetable consumption, BMI, physical activity, and alcohol use and twice the weight (1/3) for cigarette smoking (given strong associations between smoking and both CVD and cancer). Based on the distribution of the data, previous literature, and team discussions, we used the wHBS for our primary outcome. For ease of interpretation, we dichotomized the wHBS at the midpoint with 0–6 scores considered “low” and scores of 7–12 considered “high.”

**Table 1:** Components of the weighted health behavior score

Component	0 Point	1 Point	2 Points
Cigarette smoking (1/3)	Current smoker	Former smoker	Never smoked
Physical activity (1/6)	None	Some moderate or vigorous but less than ideal	> 150 min of moderate activity weekly
Body mass index (kg/m^2^) (1/6)	> 30	25–30	< 25
Alcohol intake (1/6)	≥ 2 drinks for women daily and ≥ 3 or more for men	1 drink per day for women and 2 for men	< 1 drink per day for women or 1 or less drink per day for men
Diet (1/6)	less than 3 servings* per day of fruits and/or vegetables	3–4 servings per day of fruits and/or vegetables	More than 4 servings per day of fruits and/ or vegetables

*One serving of vegetables is a half cup of cooked vegetables or a cup of raw vegetables. Examples of one serving of fruits are one banana, a medium apple, or a handful of grapes

### Key explanatory variables

We captured an individual’s perceived risk for cancer and CVD using one core question reframed for each condition separately, “*Compared with other people your age, how likely are you to get cancer (CVD in the parallel condition) in your lifetime?*” Responses used a 5-point Likert scale ranging from 0 “much less likely” to 4 “much more likely.” Lower scores reflect less perceived risk, and higher scores reflect greater levels of perceived risk. We grouped individuals who perceived themselves to be at increased risk for each condition (much more likely and more likely responses, 3 or 4) versus those who perceived themselves to be at less or equal risk than others their age (0, 1, or 2). Risk perception variables for cancer and CVD were analyzed as binary indicators with less or equal risk as to the reference.

### Covariates

To better understand associations between perceived cancer and CVD risk and health behaviors, separately, we examined the effects of sequentially adjusting for factors that may modify the relationship between perceived risk and health behaviors as described in the Health Belief Model. We considered the modifying factors of age, sex, race/ethnicity, marital status, education level, homeownership status, neighborhood, foreign-born status, health insurance (yes/no), self-reported comorbidities, perceived stress level, perceived general health, having a usual place of health care, and health information-seeking behavior (perceived self-efficacy). In multivariable models, we included covariates that differed significantly by low (0–6)- and high (7–12)-weighted HBS groups.

### Statistical analysis

Response rates for phone/web-based surveys were calculated using the number of participants that responded to the survey divided by the total sample size minus ineligible individuals, per Response Rate 1 guideline outlined by the American Association for Public Opinion Research [19]. Phone and web survey break-offs (i.e., interviews that ended before the outcome question were asked) were not counted as responses. Response rates for the in-person survey were calculated based on the number of eligible participants who completed the survey divided by the number of eligible participants who were approached at each venue over the study period.

Descriptive statistics using chi-square tests for categorical variables and analysis of covariance models for continuous variables were conducted. We then examined the distribution of the unweighted and weighted HBS. We estimated modified Poisson models with robust standard errors to assess the association between perceived cancer risk and the weighted HBS (dichotomized 0–6 vs. 7–12). We estimated both univariate and multivariable models. We repeated this approach for perceived CVD risk. In multivariable models, we adjusted for sex, race/ethnicity, foreign-born status, marital status, education, employment status, home ownership, living situation, food insecurity, perceived overall health, and chronic health conditions. We calculated prevalence ratios (PR) and 95% confidence intervals (95% CI) for all estimates. We also conducted a sensitivity analysis using a median-split wHBS (0–8 vs. 9–12). As an additional sensitivity analysis, we examined the wHBS as a continuous outcome. All analyses were conducted in SAS version 9.4 with two-sided statistical tests and α of 5%.

## Results

### Response rates

Among 15,520 attempted calls, 6,610 (42.6%) adults were eligible for participation. Of these, 1,644 surveys were completed by phone and 17 by web for a response rate of 25.2%. There were 7,733 prospective eligible adults on-site across all venues during the period of venue-based survey administration. Of these, 2,680 (35%) participants were approached and 787 surveys were completed for a response rate of 29.3% (range 20.0–51.6%).

### Participant characteristics

Among the 2,448 survey participants, mean age was 61.4 years (SD 12.9), 61% were female, 49% were Black, 30% Non-Hispanic White, 11% Hispanic, and 4% Asian (Table 2). Forty-one percent of respondents were foreign-born, 36% had a high school education or less, and 18% reported food insecurity. Hypertension (53%), arthritis (39%), and diabetes (28%) were the most reported health conditions and 74% of respondents had at 1 or more chronic health condition (diabetes, hypertension, depression, arthritis or rheumatism, cardiovascular disease). Twenty percent of respondents reported depression or anxiety and 12% reported a history of cancer. Nearly a quarter (24%) reported feeling stressed “always” or “frequently” and 26% reported their overall health as “fair” or “poor.”

**Table 2:** Characteristics of Survey Respondents overall (*n* = 2,448) and by wHBS (*n* = 2,349)

	All, *n* = 2,448	wHBS (0–6) *n* = 577	wHBS (7–12) *n* = 1772	*p* value
*Sociodemographics*				
Age				
31–40	74 (3%)	19 (3%)	52 (3%)	0.27
41–50	512 (21%)	120 (21%)	370 (21%)	
51–60	595 (24%)	158 (27%)	417 (24%)	
61–70	624 (25%)	145 (25%)	452 (26%)	
71 and above	643 (27%)	135 (23%)	481 (27%)	
Female	1,501 (61%)	314 (54%)	1,121 (63%)	< 0.001
Race				
Non-Hispanic Black	1,195 (49%)	283 (49%)	879 (50%)	< 0.001
Non-Hispanic White	725 (30%)	162 (28%)	526 (30%)	
Hispanic	277 (11%)	82 (14%)	180 (10%)	
Non-Hispanic Asian	102 (4%)	9 (2%)	89 (5%)	
Other/Unknown	149 (6%)	41 (7%)	98 (6%)	
Foreign-born	1001 (41%)	151 (26%)	821 (46%)	< 0.001
Marital status				
Married	1091 45%)	217 (38%)	841 (48%)	< 0.001
Other (divorced, widowed, separated)	717 (30%)	180 (31%)	505 (29%)	
Single, never been married	618 (25%)	180 (31%)	411 (23%)	
Education				
Less than high school	275 (11%)	65 (11%)	195 (11%)	< 0.001
High school graduate	618 (25%)	165 (29%)	424 (24%)	
Tech/vocational school/Some college	543 (22%)	165 (29%)	358 (20%)	
College graduate	996 (41%)	180 (31%)	783 (44%)	
Occupation				
Employed	924 (38%)	195 (34%)	697 (39%)	< 0.001
Retired	1,061 (44%)	229 (40%)	791 (45%)	
Unemployed	172 (7%)	64 (11%)	100 (6%)	
Other (homemaker, student, disabled)	280 (11%)	87 (15%)	178 (10%)	
Has health insurance	2,367 (97%)	554 (96%)	1,718 (97%)	0.27
Owns their own home	1,126 (46%)	221 (39%)	869 (49%)	< 0.001
Living situation				
Steady place to live	2,280 (93%)	515 (90%)	1,681 (95%)	< 0.001
Worried about losing housing	109 (4%)	37 (6%)	62 (4%)	
No steady place to live	54 (2%)	23 (4%)	27 (2%)	
Presence of food insecurity	455 (18%)	135 (24%)	299 (17%)	< 0.001
Self-perceived health				
Excellent–Very Good	918 (38%)	163 (28%)	730 (41%)	< 0.001
Good	893 (37%)	207 (36%)	644 (36%)	
Fair–Poor	625 (26%)	204 (36%)	392 (22%)	
Perceived Stress				
Always or frequently	582 (24%)	168 (29%)	386 (22%)	< 0.001
Occasionally	851 (35%)	224 (39%)	594 (34%)	
Never or rarely	1,000 (41%)	180 (31%)	783 (44%)	
Presence of ≥ 1 chronic health conditions	1,808 (74%)	472 (82%)	1,259 (71%)	< 0.001
Prior history of cancer	304 (12%)	82 (14%)	207 (12%)	0.11
*Health care access*				
Usual place of care				
Primary care doctor’s office	2,140 (87%)	483 (84%)	1,577 (89%)	< 0.001
Hospital emergency room	258 (11%)	85 (15%)	164 (9%)	< 0.001
Urgent care center	174 (7%)	45 (8%)	121 (7%)	0.43
Pharmacy or retail clinic	38 (2%)	8 (1%)	29 (2%)	0.68
Some other place^a^	105 (4%)	22 (4%)	78 (4%)	0.54
Looks up health information on their own	1,593 (65%)	351 (61%)	1,188 (67%)	0.007

^a^Includes “specialist,” “relative,” “walk-in-clinic,” and “Do not know health advice location”

### Weighted health behavior score

The wHBS score ranged from 0 to 12 with a mean of 7.9 (SD 2.2) and a median of 8 (IQR 7–9). Overall, 24.6% of respondents had low scores (0–6 points) compared to 75.4% of respondents who had scores of 7–12 (Table 3). Compared to having a high wHBS (7–12) characteristics that were significantly associated with low wHBS scores (*p* < 0.05) included male gender (47% vs 38%), Hispanic ethnicity (14% vs 10%), being born in the USA (74% vs. 54%), being single (31% vs. 23%), less educated (69% vs. 56%), not owning a home (39% vs. 49%), being food insecure (24% vs. 17%), having 1 or more chronic health conditions (82% vs. 71%), greater perceived stress (29% vs. 22%), and fair/poor overall quality of health (36% vs. 22%).

**Table 3:** Distribution of the components of the weighted health behavior score

	All
All, *n* (row %)	2,448 (100%)
Body mass index	
0 (> 30 kg/m2)	779 (32%)
1 (25–30 kg/m2)	878 (37%)
2 (< 25 kg/m2)	744 (31%)
Physical activity, weekly	
0 (None)	334 (14%)
1 (some moderate or vigorous but less than ideal)	878 (36%)
2 (> = 150-min moderate or > = 75-min vigorous activity)	1,229 (50%)
Alcohol, daily consumption	
0 (> = 2 drinks for women and > = 3 for men)	344 (14%)
1 (1 drink for women and 2 for men)	457 (19%)
2 (< 1 drink for women and < 2 drinks for men)	1,632 (67%)
Cigarette smoking	
0 (Current smoker)	263 (11%)
1 (Quit more than 12 months ago)	564 (23%)
2 (Never smoked)	1,612 (66%)
Whole fruits and/or vegetables, daily consumption	
0 (< = 2 servings)	762 (31%)
1 (3–4 servings)	1,131 (47%)
2 (> 4 servings)	529 (22%)
Health behavior score (unweighted)*	
0–5 (worst)	720 (31%)
6	466 (20%)
7	545 (23%)
8–10 (best)	618 (26%)
Health behavior score (weighted)**	
0–6 (worst)	577 (25%)
7–8	558 (24%)
9	741 (32%)
10–12 (best)	473 (20%)

*The Health Behavior Score (unweighted) is the sum of the responses for each of the five individual behaviors and ranges from 0 (does not adhere to any of the recommendations) to 10 for (fully adherent to all five recommendations)

**The weighted health behavior score assigned equal weights (1/6) for fruit and vegetable consumption, BMI, physical activity, and alcohol use and twice the weight (1/3) for cigarette smoking

### Risk perceptions and health behaviors

Sixty-six percent of respondents with low wHBS scores perceived that they were at lower risk for cancer and CVD compared to other adults their age. Cancer and CVD risk perception were minimally correlated (*r* 0.22; *p* < 0.0001).

**Fig. 1:**
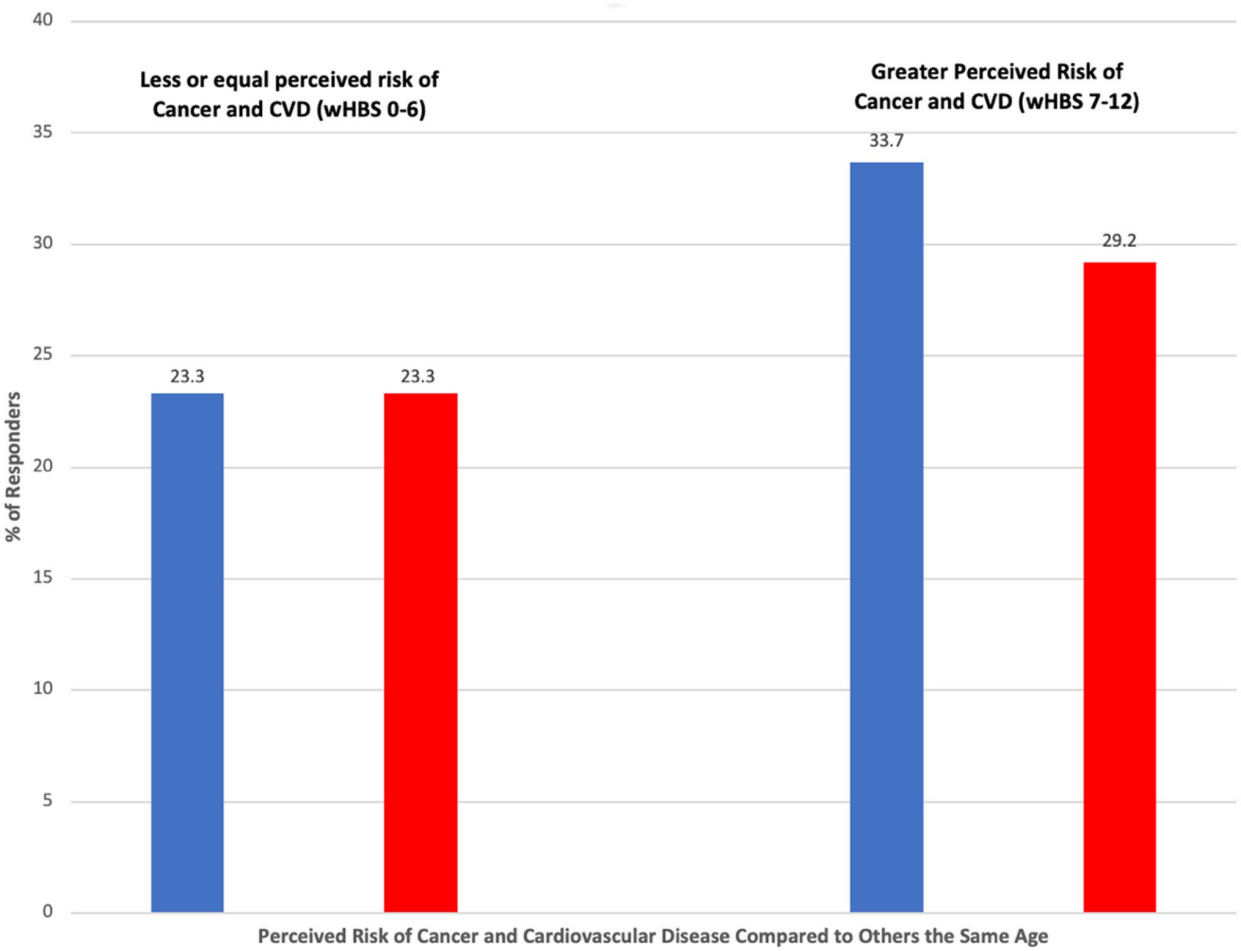
Self-perceived risk of cancer and cardiovascular disease by weighted health behavior score (wHBS). Blue bars represent cancer and red bars represent CVD. There were *n* = 1,559 individuals who reported that they perceived themselves to have a lower or equal risk of cancer and *n* = 1,497 individuals who reported that they perceived themselves to have a lower or equal risk of CVD compared to others their age. There were *n* = 252 individuals who reported that they perceived themselves to have a greater risk of cancer and *n* = 370 individuals who reported that they perceived themselves to have a greater risk of CVD compared to others their age. (Color figure online)

For the cancer risk perception and health behavior analyses (Fig. 1), we excluded 307 respondents who reported a history of cancer, 263 individuals who did not respond to the cancer risk question, and 67 who had missing responses for the health behavior questions. Among the remaining 1,811 eligible participants, 252 (13.9%) respondents reported that they were more likely to get cancer in their lifetime compared to others their age. Of these, 33.7% had a low wHBS (0–6 points). In contrast, individuals who perceived lower or equal cancer risk, 23.3%, had a low wHBS (*p* = 0.0004).

In the CVD perceived risk and health behavior analysis, we excluded 327 respondents who self-reported a history of CVD, 188 who did not respond to the CVD risk perception question, and 66 people did not complete the questions about their health behaviors (Fig. 1). As such, of the 1,867 eligible participants, 370 (19.8%) respondents reported they were more likely to have a heart attack or stroke in their lifetime compared to others their age. Among the 370 individuals who perceived themselves to be at greater CVD risk, 29.2% had a low engagement in behaviors that reduce their risk (wHBS 0–6 points). In contrast, for those who reported themselves to be at lower or equal risk of CVD, 23.3% had a low wHBS (*p* < 0.0001).

**Fig. 2:**
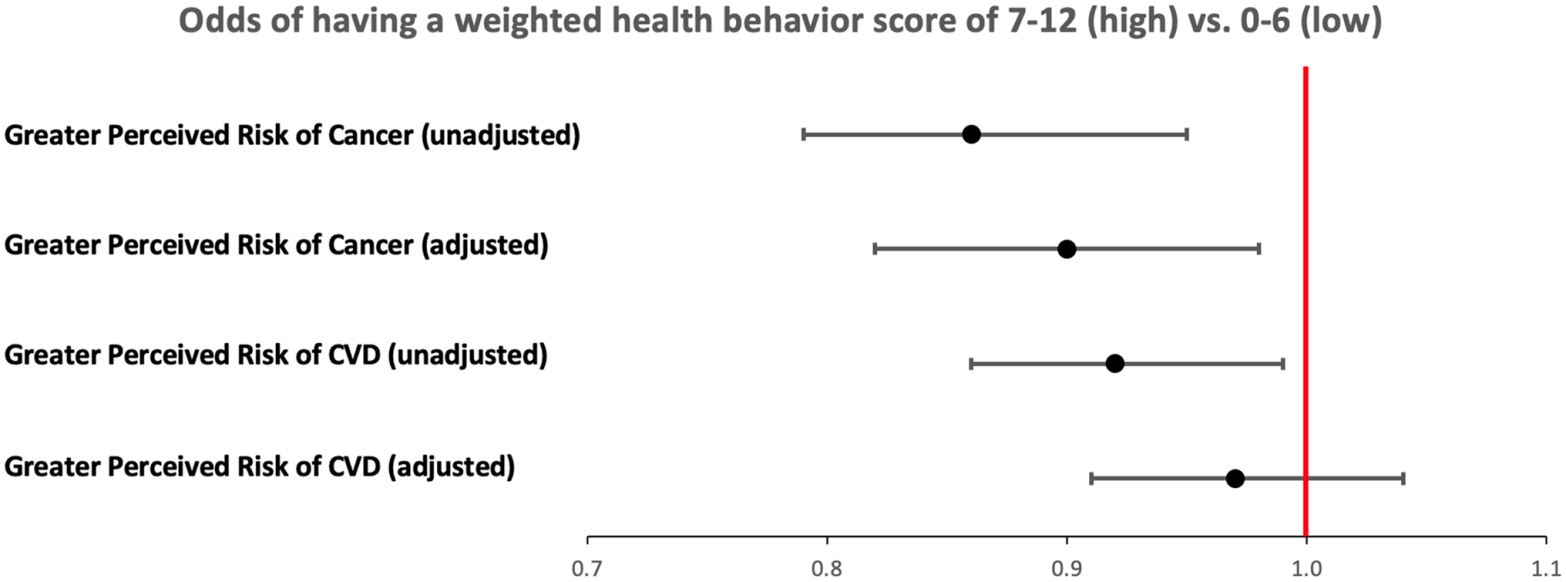
Associations between perceived risk for cancer and cardiovascular disease and weighted health behavior score^a^. ‘Much less likely,’ ‘Less likely,’ and ‘About the same’ responses collapsed to ‘Less/same.’ Greater, ‘Much more likely’ responses collapsed to ‘More.’ ^a^Modified Poisson regression modeling was used for the adjusted analysis and prevalence ratios and 95% confidence intervals are shown along the x-axis. ^b^Multivariable models adjust for sex, race/ethnicity, foreign-born status, marital status, education, employment status, home ownership, living situation, food insecurity, perceived overall health, and chronic conditions (diabetes, hypertension, depression, arthritis, or rheumatism). The “perceiver-risk-of-cancer” model includes cardiovascular disease indicator; the “perceiver-risk-of-CVD” model includes cancer and lung indicators

In unadjusted modified Poisson models (Fig. 2), we found that perceiving oneself to have greater cancer risk was associated with a 14% lower likelihood of adhering to more ideal health behaviors (wHBS score 7–12) (PR 0.86; 95% CI 0.79–0.95) (Table 4). Once potential confounders were accounted for, the effect attenuated but continued to be statistically significant (aPR 0.90; 95% CI 0.82–0.98). Similarly, we found that perceiving oneself to have more CVD risk was associated with an 8% lower likelihood of adhering to more ideal health behaviors (wHBS score 7–12) (PR 0.92; 95% CI 0.86–0.99). However, once potential confounders (the same as in the “cancer” model, except a CVD history indicator, substituted the prior cancer history indicator) were accounted for, the effect attenuated and became non-significant (aPR 0.97; 95% CI 0. 91–1.04). In a sensitivity analysis when we used a median split for the weighted HBS, the crude association between perceiving more cancer risk and the likelihood of completing more health behaviors (9–12) compared to fewer health behaviors (0–8) was greater (crude PR 0.81; 95% CI 0.68–0.96 and fully adjusted PR 0.85; 95% CI 0.72–1.00). We observed a similar pattern for CVD risk perception with a crude PR of 0.79 (95% CI 0.68–0.92) and fully adjusted PR 0.91 (95% CI 0.78–1.05). Results from the models using a continuous wHBS as the outcome were nearly identical (results now shown).

**Table 4:** Associations between perceived risk for cancer and cardiovascular disease and weighted health behavior score (wHBS)

	Perceived cancer risk		Perceived CVD risk	
	Crude model (95% CI)	Fully adjusted (95% CI)	Crude model (95% CI)	Fully adjusted (95% CI)
wHBS dichotomized (0–6, 7–12)	0.86 (0.79–0.95)	0.90 (0.82–0.98)	0.92 (0.86–0.99)	0.97 (0.90–1.04)

The models assessed the likelihood of adhering to more ideal health behaviors (higher wHBS score). Exposure is perceived risk of cancer/CVD with reference “less or same risk.” Fully adjusted includes sex, race/ethnicity, foreign-born status, marital status, education, employment status, home ownership, living situation, food insecurity, perceived overall health, and chronic health conditions

### Discussion

To our knowledge, this is among the first studies to evaluate the association between perceived risk for cancer alongside CVD and engagement in modifiable lifestyle behaviors that reduce morbidity and mortality from both health conditions. Overall, nearly a quarter (24.6%) of respondents were not engaged in risk-reducing behaviors. Interestingly, among these respondents, more than half (66.3%) perceived that their risk for cancer or CVD was lower than their age counterparts despite their low engagement in risk-reducing health behaviors. This finding is consistent with previous studies in which the general public tends to be optimistic about their risk for chronic health conditions [20, 21]. Perceiving oneself to have high risk can be anxiety provoking, suggesting that optimistic biases in risk perceptions (i.e., perceiving one’s risk to be lower than it is) might be health promoting [22].

Among respondents with a low health behavior score (0–6) a slightly higher proportion (29.2%) perceived they were at greater risk for CVD compared to their age counterparts than cancer (23.3%). Similarly, despite low engagement in risk-reducing behaviors a greater proportion of respondents (33.7%) perceived they were at less or the same risk for cancer compared to their age counterparts in comparison to their self-perceived risk for CVD (23.3%). We found a low association between respondents’ perceived risk for cancer and CVD, suggesting that respondents likely do not view the risk factors for these two conditions the same.

We initially hypothesized that individuals with a higher perceived risk of cancer or CVD would report engaging in more beneficial health behaviors. However, we observed the opposite association for cancer, as individuals who perceived themselves to be at greater risk were less likely to engage in healthy behaviors in both unadjusted and adjusted models. It is possible that those who do not engage in risk-reducing health behaviors appropriately perceive themselves to be at higher risk for cancer. It is also possible that perceiving oneself to be at increased risk for cancer leads an individual to want to engage in fewer healthy behaviors. Due to the nature of the cross-sectional survey, the direction of any association is unclear. A longitudinal study to understand the directionality of this association is warranted. Despite this limitation, our study identifies a self-reported measure that can be utilized to identify a subgroup of the population for whom a multi-level behavior change intervention that addresses risk perception, in addition to other key environmental and political factors could lead to a reduction in shared risks between CVD and cancer. We acknowledge that individuals, whose attitudes, beliefs, and habits behavioral treatments target, are embedded in a complex system that either promulgates or discourages CVD and cancer risk behaviors. The sociocultural context in which individuals live conveys norms, models, reinforcement, and inclusion when behaviors match expectations. However, on a large scale, the physical environment and public policies establish defaults and options that facilitate or thwart healthy choices. In spite of these external barriers there remains a subset of the population who are able to make key behavioral changes, hence future interventions are most relevant for those subgroups that are at risk.

Our findings for CVD demonstrated no significant association between risk perception and healthy behaviors once we accounted for confounders. Unlike previous studies [23–25] that used the HINTS risk perceptions questions, we excluded individuals who had a history of the condition from each respective analysis. This resulted in a reduction of our overall sample size for each respective question and thus may have impacted our ability to detect a difference in the multivariable model.

Although a significant burden of cancer (42% of incident cases and 45% of deaths) [26] and CVD (26% of deaths) [27] in the USA can be traced to modifiable health behaviors, cardiology, and oncology scientific societies have generally made separate and uncoordinated efforts at promoting the primary or secondary prevention of these two conditions. In a recent analysis by Lau et al. using data from the Framingham Heart Study, we find that individuals with a 10-year atherosclerotic cardiovascular disease (ASCVD) risk of 20% or higher were more than three times as likely as those with a 10-year ASCVD risk of 5% or lower to develop any cancer.

If health care is to implement integrated prevention strategies for cancer and CVD, there will need to be a shift away from disease-centric guidelines. Furthermore, successful strategies must account for complex decisions most adults face in weighing the risks and benefits of multiple competing recommendations and guidelines from professional societies and government agencies. Risk calculators likely have a less dramatic impact on subgroups whose engagement in risk-reducing behaviors is low. Behavioral science research demonstrates that subgroups may be more responsive to practices and interventions that highlight social comparisons and social identities [28], mitigate defensiveness [29], and recognize fatalistic perspectives. Our study sheds useful light on some of the individuals and/or combined characteristics of subgroups (i.e., men, adults never married, adults with less education) less likely to engage in these ideal health behaviors. Additionally, future interventions will need to account for fundamental questions about how to maximize the cumulative change in multiple health behaviors while keeping negative consequences to a minimum. One classic example [30] of unintended consequences is the typical post-cessation weight gain that occurs in individuals who quit tobacco smoking [31].

While our study is at risk for non-response bias due to an average 25–29% response rate, there is evidence that surveys with low-response rates are not necessarily low in validity [32]. In work conducted by Keeter et al. we find a comparison between two survey methodologies whereby one resulted in a 25% response rate and the other a 50% response rate. However, in 77 out of 84 comparisons, the two surveys yielded results that were statistically indistinguishable [33]. Furthermore, our response rate is comparable and, in some cases, better than similar survey studies [25]. Measurement error and misclassification in self-report, especially for health behaviors, are well known. Furthermore, unmeasured confounders that may impact risk perception and factors such as family history were not accounted for. Additionally, our sample size was not sufficient for a subgroup analysis. Finally, there may be limited external validity of the findings reported here, as we included only Brooklyn residents.

## Conclusion

Our study supports the existing evidence that a paradigm shift in cardiology and oncology is needed. Guidelines and recommendations should account for the fact that the general population does not make health decisions in separate silos. Innovative multi-behavior change interventions tailored to subgroups with the lowest engagement in shared risk-reducing behaviors between cancer and CVD have the potential to have the greatest impact on the health of the population locally and globally.

## Supplementary Information

Below is the link to the electronic supplementary material.

Supplementary file1 (DOCX 18 kb)

## Data Availability

The datasets generated during and/or analyzed during the current study are available from the corresponding author on reasonable request.

## Code availability

N/A.
