# Supplementary material for: Cancer and cardiovascular-related perceived risk in a diverse cancer center catchment area
Source: Cancer Causes Control. 2022 Mar 11;33(5):759–68. doi: 10.1007/s10552-022-01560-3 (PMC8913330; doi:10.1007/s10552-022-01560-3)
Supplement: Supplementary file 1 — Supplementary file1 (DOCX 18 kb) [file 10552_2022_1560_MOESM1_ESM.docx]

**Supplementary Materials**

**Table 1: Response rate for venue-based survey sampling**

| **Venue** | **Estimated # of people at venue (all ages)** | **# Of approaches with eligible participants** | **# Of refusals/not interested** | **# Of surveys completed** | **Response rate** |
| --- | --- | --- | --- | --- | --- |
| Clinical Practice (group or solo) | 2059 | 1137 | 824 | 228 | 20.0% |
| Faith based organizations | 795 | 180 | 93 | 93 | 51.6% |
| Public Library | 3260 | 748 | 429 | 184 | 24.6% |
| Service provider (food pantry, etc.) | 1454 | 568 | 183 | 263 | 46.3% |
| Schools | 165 | 47 | 28 | 19 | 40.2% |
| Total | 7733 | 2680 | 1557 | 787 | 29.3% |

**Table 2:** Demographic and socioeconomic characteristics of CARBS survey respondents and data from the American Community Survey 2015-2019

|  | ACS | CARBS | ACS | | CARBS | | ACS | | CARBS | ACS | CARBS |
| --- | --- | --- | --- | --- | --- | --- | --- | --- | --- | --- | --- |
|  | **Bed Stuy** | **Bed Stuy** | **Crown N.** | **Crown S.** | **Crown Heights** | | **Flatbush** | **E. Flatbush** | **Flatbush & Midwood** | **Coney Island** | **Coney Island** |
| Population | 0.14 * | 519 | 0.13* | 0.11* | 758 | | 0.16* | 0.14* | 617 | 0.12* | 554 |
| **Race/ Ethnicity** |  |  |  |  |  | |  |  |  |  |  |
| Asian,% | 3.8 | 1 | 4.1 | 2.1 | 1.3 | | 9.9 | 1.3 | 2.6 | 12.8 | 12.8 |
| Hispanic,% | 18.1 | 12.7 | 12.9 | 9.3 | 9.1 | | 15.4 | 7.7 | 9.9 | 14.8 | 14.6 |
| Non-Hispanic White,% | 23 | 8.3 | 27.2 | 27 | 16.9 | | 39.6 | 3.4 | 37.6 | 57.7 | 58.1 |
| Non-Hispanic Black,% | 52.5 | 72.3 | 51.7 | 58.7 | 64.9 | | 32.2 | 85.6 | 43.3 | 11.4 | 11 |
| Other | 2.5 | 5.8 | 4 | 2.9 | 7.8 | | 2.8 | 1.8 | 6.6 | 3.1 | 3.4 |
| **Place of Birth** |  |  |  |  |  | |  |  |  |  |  |
| Foreign Born, % | 19.5 | 30.6 | 27 | 40.1 | 45.9 | | 41.4 | 51.6 | 48.6 | 53.5 | 35 |
| **Social determinants of Health** |  |  |  |  |  | |  |  |  |  |  |
| No health insurance, % | 7.3 | 4.2 | 7.2 | 7.4 | 4.1 | | 8.7 | 8.5 | 2.8 | 7.4 | 2 |
| With public health insurance,% |  |  |  |  |  |  |  |  |  |  |  |
|  | 48 | 52.1 | 40.7 | 43.8 | 53.5 | | 46.6 | 44.3 | 44.6 | 56.4 | 56.8 |

*: million **Bed Stuy**= Bedford-Stuyvesant,Brooklyn; **Crown N**= Crown Heights North, Brooklyn; **Crown S**=Crown Heights South, Brooklyn; **Crown H**=Crown Heights, Brooklyn; **Flatbush**, Brooklyn; **E.Flatbush**= East Flatbush, Brooklyn;
